# De Novo *EGFR* ‐ *ALK* and *EGFR* ‐ *ROS1* Co‐Mutations in NSCLC: Clinical Characteristics, Molecular Profiling, and Treatment Outcomes From a Retrospective Analysis

**DOI:** 10.1002/cam4.71084

**Published:** 2025-07-29

**Authors:** Lili Shen, Hongyu Deng, Wei Liao, Qiang Gu, Lingling Ma, Qingming Jiang, Kaihua Liu

**Affiliations:** ^1^ Department of Pathology Chongqing University Cancer Hospital China; ^2^ Chongqing Key Laboratory for Intelligent Oncology in Breast Cancer Chongqing University Cancer Hospital China; ^3^ The Affiliated Cancer Hospital of Xiangya School of Medicine Central South University/Hunan Cancer Hospital Changsha China; ^4^ Institute of Cardiovascular Surgery Xin Qiao Hospital, Second Affiliated Hospital of the Army Military Medical University Chongqing China; ^5^ Nanjing Geneseeq Technology Inc. Nanjing Jiangsu China

**Keywords:** *de novo* mutation, *EGFR*‐*ALK* co‐mutation, *EGFR*‐*ROS1* co‐mutation, non‐small cell lung cancer, targeted therapy

## Abstract

**Background:**

*De novo* epidermal growth factor receptor‐anaplastic lymphoma kinase (*EGFR*‐*ALK*) and *EGFR*‐ROS proto‐oncogene 1 (*EGFR*‐*ROS1*) co‐mutations in non‐small‐cell lung cancer (NSCLC), conditions traditionally considered mutually exclusive. We present the first large‑scale analysis of their clinical and genomic profiles.

**Methods:**

We identified 26 patients with *EGFR*‐*ALK* (*n* = 20) or *EGFR*‐*ROS1* (*n* = 6) co‐mutations from two institutions and compared them with cohorts of *EGFR*‐only, *ALK*‐only, *ROS1*‐only, and non‐co‐mutated (NC) controls. Additionally, we validated findings in 66 published co‐mutation cases through a literature review (2010–2023).

**Results:**

The co‐mutation frequencies were 0.36% for *EGFR*‐*ALK* and 0.11% for *EGFR*‐*ROS1*. *EGFR*‐*ALK*co‐mutations were more commonly diagnosed at earlier stages (50.0% stage 0‐II vs. 22.6% in *ALK*‐only, *p* = 0.03). Patients with *EGFR*‐*ALK* co‐mutations were older than those with *ALK*‐only mutations (≥ 60 years: 60.0% vs. 24.5% in *ALK*‐only, *p* = 0.01), a trend validated in external pooled cases (51.9% vs. 24.5%, *p* = 0.01). All *EGFR*‐*ROS1*cases were never‐smokers and predominantly female (66.7%), a trend consistent with external pooled cases. Co‐mutated tumors were enriched for *EGFR* exon 19 deletion (19del, 60.0% vs. 42.2% *EGFR*‐only) and depleted for L858R. Additionally, 80.0% of *EGFR*‐*ALK* cases harbored the *EML4*‐*ALK*V3. Non‐smokers exhibited superior overall survival (OS) in both cases (internal *p* = 0.002, external pooled cases *p* = 0.03). Among 10 advanced‐stage patients with sufficient clinical follow‐up, two had received dual‐targeted TKI therapies. Both patients tolerated dual‐targeted TKI therapies well, with one requiring dose adjustment due to initial toxicity and subsequently achieving an overall survival exceeding 51 months; however, limited sample size precludes definitive conclusions regarding efficacy.

**Conclusion:**

*De novo* co‐mutations represent a distinct NSCLC subset with unique clinical and genomic features, and dual‐targeted therapy shows promise as a strategy that warrants evaluation in prospective studies.

## 1 Introduction

Epidermal growth factor receptor (*EGFR*) mutations, as well as rearrangements in anaplastic lymphoma kinase (*ALK*) and ROS proto‐oncogene 1 (*ROS1*), are well‐established oncogenic drivers in non‐small‐cell lung cancer (NSCLC). Traditionally, these alterations were considered mutually exclusive, with concurrent mutations primarily reported in the context of acquired resistance to EGFR tyrosine kinase inhibitors (TKIs) [1, 2]. However, advances in sensitive detection techniques such as next‐generation sequencing (NGS) have revealed that these mutations can coexist at diagnosis, thereby challenging previous paradigms and underscoring the need for comprehensive studies to better define their clinical and molecular characteristics [3, 4]. Most previous reports on *EGFR*‐*ALK* or *EGFR*‐*ROS1* co‐mutations have been limited to isolated case reports or small case series [1, 2, 3, 4, 5, 6, 7], highlighting the necessity for a more comprehensive analysis.

In our study, we present the first large‐scale investigation of *de novo EGFR*‐*ALK* and *EGFR*‐*ROS1* co‐mutations. We integrated data from our institution, collected between September 2019 and July 2024, and validated these findings with pooled cases derived from published literature. This dual‐cohort design enhances the generalizability of our findings and helps to address the temporal and stage‐related biases typical of rare mutation studies. By focusing on patients with these co‐mutations detected exclusively prior to any EGFR‐TKI treatment, we aim to systematically evaluate their clinical characteristics, prognostic factors, and treatment outcomes, ultimately paving the way for more personalized therapeutic strategies.

## 2 Methods

### 2.1 Study Design and Patient Cohorts

We retrospectively screened 5539 consecutive Chinese NSCLC cases diagnosed at two centers—Chongqing Cancer Hospital and Hunan Cancer Hospital—between September 2019 and July 2024. Genomic profiling across the study population was performed using either NGS or multiplex PCR. From this bi‐institutional dataset, we identified patients with *de novo EGFR*‐*ALK* or *EGFR*‐*ROS1* co‐mutations.

For comparison, we generated a non‐co‐mutated (NC) control group that is entirely drawn from the same cohort of 5539 cases. In line with institutional policies and data access constraints, this control group was curated from an institutional database at Chongqing Cancer Hospital, covering a pre‐approved period from 2021 to 2023, during which comprehensive clinical and molecular data were consistently available. The NC group includes all cases from the original cohort except those with *EGFR*‐*ALK*/*ROS1* co‐mutations (*n* = 2924). Among these, 1398 patients underwent NGS, allowing further stratification into subgroups with only *EGFR* mutations (*n* = 719), only *ALK* fusions (*n* = 53), or only *ROS1* fusions (*n* = 16). In the NC cohort, 47.8% of patients underwent NGS testing and 52.2% received multiplex PCR, whereas in the co‐mutation cohort, 38.5% underwent NGS and 61.5% received multiplex PCR. There was no significant difference in the distribution of testing methods between the two cohorts (*p* = 0.43, Figure S1A). Among patients tested by NGS, the proportion receiving the 14‐gene panel versus the 196‐gene panel was similar between the NC (74.7% vs. 25.3%) and co‐mutation (80.0% vs. 20.0%) cohorts (*p* = 0.99, Figure S1B).

For external validation, we assembled a pooled cohort of cases (*n* = 66) via a literature review (2010–2024), comprising 58 *EGFR*‐*ALK* and eight *EGFR*‐*ROS1* co‐mutations that met the inclusion criteria of *de novo co*‐mutations confirmed through molecular testing prior to EGFR‐TKI treatment initiation. Overall survival (OS) was defined as the time from diagnosis to death or last follow‐up, whereas progression‐free survival (PFS) was defined as the time from initiation of a given line of therapy to documented disease progression or death. Since OS data were unavailable for the pooled literature cases, we evaluated the summed PFS across multiple lines of therapy to further assess prognostic impact.

### 2.2 Molecular Testing

Archived formalin‐fixed paraffin‐embedded tumor blocks/slides, frozen fresh tissues were used for tumor genotyping. Tumor tissues were first inspected by pathologists to ensure that they contained at least 20% tumor content and sufficient samples were available for testing. Molecular profiling was performed using NGS with either a 14‐gene or 196‐gene panel (Nanjing Geneseeq Technology), following protocols outlined previously [8] and detailed in the Supporting Information. Amplification refractory mutation system polymerase chain reaction (ARMS‐PCR) was conducted using the AmoyDx 3‐Gene Assay, which detects *EGFR* mutations, as well as *ALK* and *ROS1* fusions. The specific gene lists for the 14‐gene and 196‐gene NGS panels, as well as the AmoyDx 3‐Gene Assay, are provided in Table S1. Quality controls included no‐template controls and 10% of samples tested in duplicate.

### 2.3 Statistical Analysis

Statistical analysis was performed using R version 4.0.4. Categorical variables were analyzed using chi‐square or Fisher's exact tests (for *n* < 10). OS was defined as the time from diagnosis to death or last follow‐up and was estimated using Kaplan–Meier survival curves. The prognostic impact of clinical variables, including age, smoking status, and mutation subtype, was assessed using Cox proportional hazards regression, with *p* < 0.05 considered statistically significant.

## 3 Results

### 3.1 Patient Demographics and Clinical Features

In a two‐center NSCLC cohort diagnosed between September 2019 and July 2024 (*n* = 5539), *de novo EGFR*‐*ALK* co‐mutations were detected in 0.36% of cases (20/5539), whereas *EGFR*‐*ROS1* co‐mutations were even rarer at 0.11% (6/5539) (Table 1). Baseline clinical characteristics for the 26 patients with *de novo* co‐mutations are detailed in Table 1 and Table S2.

**TABLE 1:** Clinical features of NSCLC with *EGFR‐ALK/ROS1*co‐mutations vs. single mutations and NC group.

Characteristics	Mutation subtypes						Statistics (*p*‐value)						
	NC (*n* = 2924)	*EGFR‐only* (*n* = 709)	*ALK‐only* (*n* = 53)	*ROS1‐only* (*n* = 16)	*EGFR‐ALK* (*n* = 20)	*EGFR‐ROS1* (*n* = 6)	NC	*EGFR‐*only	*ALK‐*only		NC	*EGFR‐*only	*ROS1‐*only
							vs. *EGFR‐ALK*				vs. *EGFR‐ROS1*		
**Gender, *n (%)***							0.62	0.77	0.66		0.43	1.00	1.00
female	1373 (47.0%)	431 (60.8%)	34 (64.2%)	10 (62.5%)	11 (55.0%)	4 (66.7%)							
male	1551 (53.0%)	278 (39.2%)	19 (35.8%)	6 (37.5%)	9 (45.0%)	2 (33.3%)							
**Age, *n (%)***							**0.32**	**0.31**	**0.01**		1.00	1.00	0.62
age > 60	1356 (46.4%)	326 (46.0%)	**13 (24.5%)**	5 (31.3%)	**12 (60.0%)**	3 (50.0%)							
age ≤ 60	1568 (53.6%)	383 (54.0%)	**40 (75.5%)**	11 (68.8%)	**8 (40.0%)**	3 (50.0%)							
**TNM Stage, *n (%)***													
0	**17 (0.6%)**	4 (0.6%)	**0 (0%)**	0 (0%)	**1 (5.0%)**	0 (0%)	**0.03**	**0.05**	**0.03**		0.43	0.43	1.00
I‐II	**1119 (38.3%)**	274 (38.6%)	**12 (22.6%)**	2 (12.5%)	**9 (45.0%)**	1 (16.7%)							
III‐IV	**1788 (61.1%)**	431 (60.8%)	**41 (77.4%)**	14 (87.5%)	**10 (50.0%)**	5 (83.3%)							
**Smoking History, *n (%*)**							0.58	0.80	0.55		**0.04**	**0.19**	**0.53**
no	**1648 (56.4%)**	498 (70.2%)	40 (75.5%)	**13 (81.3%)**	13 (65.0%)	**6 (100%)**							
yes	**1276 (43.6%)**	211 (29.8%)	13 (24.5%)	**3 (18.8%)**	7 (35.0%)	**0 (0%)**							
**Pathology, *n (%)***													
adenocarcinoma	2528 (86.5%)	20 (2.8%)	49 (92.5%)	14 (87.5%)	20 (100%)	6 (100%)	0.21	0.74	NA		1.00	1.00	1.00
squamous	393 (13.4%)	1 (0.1%)	4 (7.5%)	2 (12.5%)	0 (0%)	0 (0%)							
other	3 (0.1%)	688 (97.0%)	0 (0%)	0 (0%)	0 (0%)	0 (0%)							

*Note:* NC: non‐co‐mutated, NSCLC without *EGFR‐ALK* or *EGFR‐ROS1* co‐mutations. *EGFR*‐only: *EGFR* mutation only; *ALK*‐only: *ALK* fusion only; *ROS1*‐only: *ROS1* fusion only. *EGFR‐ALK*: *EGFR* and *ALK* co‐mutation; *EGFR‐ROS1*: *EGFR* and *ROS1* co‐mutation. *EGFR*‐only, *ALK*‐only, and *ROS1*‐only were identified by NGS from a subset of 1398 patients within the NC group. *P*‐values were calculated by Chi‐square test (*EGFR‐ALK* comparisons) and Fisher's exact test (*EGFR‐ROS1* comparisons).

Within the internal co‐mutation cohort, 50.0% of patients with *EGFR*‐*ALK* co‐mutations were diagnosed at early stages (stages 0‐II), a proportion significantly higher than that observed in the *ALK*‐only (22.6%, *p* = 0.03), *EGFR*‐only (39.2%, *p* = 0.05), and NC cohorts (38.9%, *p* = 0.03). Furthermore, 60.0% of the *EGFR*‐*ALK* group were aged ≥ 60 years, compared to 24.5% in the *ALK*‐only group (*p* = 0.01, Table 1). In the *EGFR*‐*ROS1* cohort, all patients were never‐smokers, a rate significantly higher than the 56.4% seen in the NC group (*p* = 0.04, Table 1), and 66.7% were female.

To validate these findings, we further analyzed 66 cases with *EGFR* *‐ALK* and *EGFR*‐*ROS1* co‐mutations pooled from published literature (Tables S3–, S4). Consistent with our internal cohort, external pooled *EGFR*‐*ALK* cases confirmed the age trend, with 51.9% of patients in the pooled *EGFR*‐*ALK* co‐mutation cases aged ≥ 60 years versus 24.5% in the *ALK*‐only group (*p* < 0.01, Table S5). However, the enrichment of early‐stage cases in our internal co‐mutated cohort was not observed in the pooled cases, likely due to historical biases in the literature where molecular testing has been predominantly conducted in advanced diseases. Despite this difference in stage distribution, the age association remained consistent in the pooled cohort. The older‐age association in *EGFR*‐*ALK* and female never‐smoker predominance in *EGFR*‐*ROS1* co‐mutations remained robust across internal and external cohorts (Table S5) [4, 6, 9].

### 3.2 Molecular Subtyping

**FIGURE 1:**
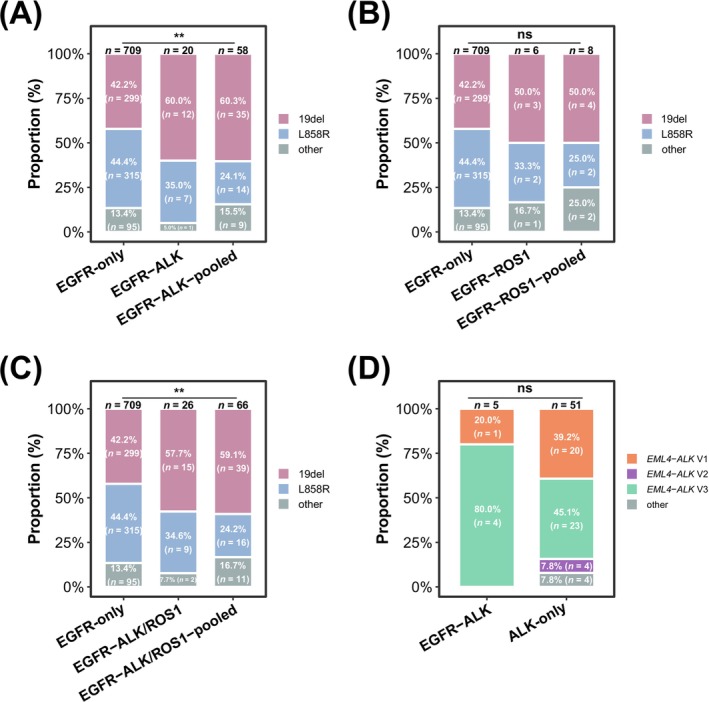
**Molecular subtyping of *EGFR* and *ALK*/*ROS1* alterations** . (A) Distribution of *EGFR* mutation subtypes among internal *de novo EGFR*‐*ALK* co‐mutated patients, *EGFR*‐only cases, and pooled previously reported *EGFR*‐*ALK* cases. (B) Distribution of *EGFR* mutation subtypes among internal *de novo EGFR*‐*ROS1* co‐mutated patients, *EGFR*‐only cases, and pooled previously reported *EGFR*‐*ROS1* cases. (C) Distribution of *EGFR* mutation subtypes among internal *de novo EGFR*‐*ALK*/*ROS1* co‐mutated cases, *EGFR*‐only patients, and pooled previously reported *EGFR*‐*ALK*/*ROS1* cases. (D) Distribution of *ALK* fusion subtypes between internal *de novo EGFR*‐*ALK* co‐mutated patients and internal *ALK*‐only patients. Percentages and case numbers are indicated within each bar segment. EGFR, epidermal growth factor receptor; ALK, anaplastic lymphoma kinase; ROS1, ROS proto‐oncogene 1; NSCLC, non‐small cell lung cancer.

Molecular subtyping revealed distinct mutation profiles among the groups (Figure 1). Exon 19 deletions (19dels) were detected in 60.0% of *EGFR*‐*ALK* cases and 50.0% of *EGFR*‐*ROS1* cases, higher than the 42.2% observed in *EGFR*‐only tumors. The difference between the *EGFR*‐*ALK* group and the *EGFR*‐only group was statistically significant (*p* < 0.01, Figure 1A). This enrichment was externally validated for *EGFR*‐*ALK* (60.3% vs. 42.2%, Figure 1A) and for *EGFR*‐*ROS1* (50.0% vs. 42.2%, Figure 1B). Conversely, the L858R mutation was observed in only 35.0% of *EGFR*‐*ALK* and 33.3% of *EGFR*‐*ROS1* cases, compared to 44.4% in *EGFR*‐only tumors. External validation confirmed reduced L858R prevalence in *EGFR*‐*ALK* (24.1% vs. 44.4%, Figure 1A) and for *EGFR*‐*ROS1* (25.0% vs. 44.4%, Figure 1B).

When the *EGFR*‐*ALK* and *EGFR*‐*ROS1* groups were combined and compared with the *EGFR*‐only group, the co‐mutated cases displayed significantly higher rates of 19dels and lower rates of L858R mutations, especially in the external validation cohort (*p* < 0.01, Figure 1C). Notably, among the five *EGFR*‐*ALK* cases analyzed by NGS, 80.0% harbored the *EML4*‐*ALK* V3 variant—a frequency significantly higher than the 45.1% observed in *ALK*‐only patients (Figure 1D).

### 3.3 Survival Analysis

Survival data were available for 17 of the co‐mutated patients in the internal cohort, with a median follow‐up time of 34.0 months (95% CI: 10.0‐not reached, Figure S2A). For pooled literature cases, PFS data summed across multiple treatment lines were collected for 19 patients as a surrogate for prognosis. No significant prognostic difference was found between the *EGFR*‐*ALK* and *EGFR*‐*ROS1* cohorts (HR = 0.76, 95% CI: 0.08–7.02, *p* = 0.81, Figure S2B). Notably, smoking status emerged as a significant predictor of survival in both internal and external cohorts. Specifically, smokers had a significantly higher risk of death compared with non‐smokers internally (HR = 9.92, *p* = 0.02) and externally (HR = 4.72, *p* = 0.03, Table S6).

**FIGURE 2:**
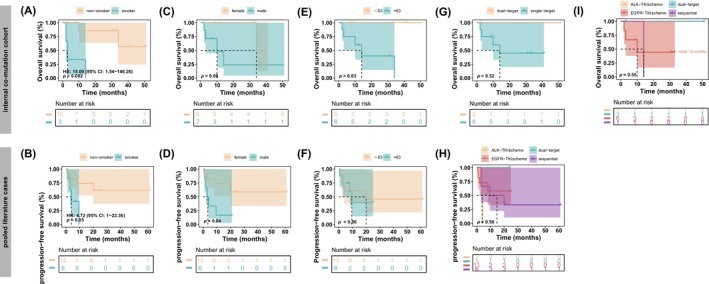
Survival analysis of NSCLC patients with *de novo* co‐mutations. Kaplan–Meier survival analyses evaluating OS and summed PFS in NSCLC patients with *de novo EGFR*‐*ALK* or *EGFR*‐*ROS1* co‐mutation, stratified by (A, B) smoking status, (C, D) gender, (E, F) age (≤ 63 vs. > 63 years), (G, H) treatment approach, and (I) treatment approach with detailed single‐target therapy. Data from the internal co‐mutation cohort (panels A, C, E, G, I) are shown in the upper panel, and pooled literature cases from previously published reports are shown in the bottom panel (panels B, D, F, H). For treatment stratification, “multi‐target” and “single‐target” refer to therapies directed against both or a single oncogenic driver, respectively; “EGFR‐TKI ± chemo/RT” represents EGFR‐TKI with or without chemotherapy and/or radiotherapy; and “sequential TKI” indicates sequential administration of TKIs targeting different oncogenic drivers. HRs and 95% CIs were estimated using Cox proportional hazards regression, and *p*‐values were obtained from log‐rank tests. NSCLC, non‐small cell lung cancer; OS, overall survival; PFS, progression‐free survival; EGFR, epidermal growth factor receptor; ALK, anaplastic lymphoma kinase; ROS1, ROS proto‐oncogene 1; TKI, tyrosine kinase inhibitor; chemo, chemotherapy; RT, radiotherapy; HR, hazard ratio; CI, confidence interval.

To minimize potential confounding due to population bias, as disease stage itself can significantly impact prognosis, we focused our survival analyses on late‐stage (stages III and IV) patients. Consistent with our findings in the entire cohort, non‐smokers remained significantly associated with improved OS compared with smokers in both datasets of late‐stage patients (*p* = 0.002 in the co‐mutation cohort; *p* = 0.03 in pooled literature cases, Figure 2A,B and Table S6). Although gender differences did not reach statistical significance in our co‐mutation cohort (*p* = 0.08, Figure 2C and Table S6), pooled literature data indicated that females had a significantly better prognosis than males (HR = 3.70, 95% CI: 0.98–13.97, *p* = 0.04, Figure 2D and Table S6). Moreover, patients aged ≤ 63 years exhibited better OS than those aged > 63 in the internal co‐mutation cohort (*p* = 0.03, Figure 2E), whereas this association was not observed in the pooled literature data (*p* = 0.56, Figure 2F).

### 3.4 Treatment Outcomes and Safety

Out of these 17 patients with survival outcomes, detailed treatment data were available for 10 advanced‐stage cases. Among these, two patients were treated with dual‐targeted TKI therapies. Patient P13 with the *EGFR*‐*ALK* co‐mutations was treated with dacomitinib and alectinib and exhibited favorable clinical outcomes with an OS exceeding 51 months. Patient P24 carrying the *EGFR*‐*ROS1* co‐mutation received gefitinib and crizotinib combination therapy, achieving stable disease at 3 m following treatment. However, follow‐up was subsequently lost for this patient.

In terms of treatment‐related toxicity, patient P13 experienced severe cutaneous toxicity during the early phase of dual therapy, necessitating a reduction of the dacomitinib dosage to 15 mg daily. This adjustment successfully alleviated the symptoms, and the dosage was later intermittently increased to 30 mg daily without causing further severe adverse reactions. On the other hand, patient P24 tolerated the combination of gefitinib and crizotinib without notable adverse effects.

Although both patients tolerated the dual‐targeted therapy well, the limited sample size prevents drawing definitive conclusions regarding the efficacy and safety of such treatments. In contrast, patients who underwent sequential or monotherapy approaches demonstrated varied outcomes. One patient treated with osimertinib followed by alectinib survived for 14 months, whereas those receiving EGFR‐TKI monotherapy (with or without additional chemotherapy/radiotherapy) had a median OS of approximately 10 m (Figure 2F). Survival analysis (Figure 2F–H) suggested a potential trend favoring dual‐targeted TKI therapy. However, these differences were not statistically significant. Due to the small sample size and limited safety data, these preliminary observations should be interpreted with caution. This underscores the necessity for prospective clinical trials to thoroughly assess the therapeutic efficacy and safety of dual‐targeted TKI strategies in NSCLC patients with *EGFR*‐*ALK* or *EGFR*‐*ROS1* co‐mutations.

## 4 Discussion

Our study characterizes the unique molecular profiles and treatment challenges of *de novo EGFR*‐*ALK* and *EGFR*‐*ROS1* co‐mutated NSCLC, a rare yet clinically significant subgroup. Both internal analysis and external validation confirm that these co‐mutations define distinct biological entities with unique demographic and molecular features.

### 4.1 Clinicopathological Features

In our internal cohort, *EGFR*‐*ALK* co‐mutations were more common in older patients (60.0% aged ≥ 60), a distribution contrasting with the typically younger demographic observed in *ALK*‐rearranged NSCLC. This finding is supported by pooled literature cases, where 51.9% of patients were aged ≥ 60 years, compared to 24.5% in the *ALK*‐only group (*p* = 0.01). This suggests age‐dependent oncogenic synergism, potentially influenced by genomic instability or environmental carcinogen exposure. Interestingly, although our cohort shows a high proportion of early‐stage diagnoses, the pooled literature cases did not display a similar concentration of early‐stage cases. This discrepancy may be due to the pooled literature cases collected earlier (predominantly in 2010–2016); pre‐2019 guidelines restricted molecular profiling to advanced NSCLC, disproportionately capturing metastatic co‐mutations in external cohorts.

*EGFR*‐*ROS1* co‐mutations were observed exclusively in never‐smokers and predominantly in females (66.7%), a pattern consistent with findings from the pooled literature cases [4, 6, 9].

### 4.2 Molecular Drivers

At the molecular level, co‐mutation groups exhibited a higher prevalence of EGFR exon 19 deletions than *EGFR*‐only tumors. At the same time, the L858R mutation was less frequent in both internal and external datasets. This suggests that exon 19 is prone to unstable, subclonal, and complex deletions. This genomic fragility may theoretically predispose tumors to secondary rearrangements like *ALK* or *ROS1* [10]. Notably, 80.0% of *EGFR*‐*ALK* cases harbored the *EML4*‐*ALK* V3 variant, a rate double that seen in *ALK*‐only cohorts (45.1%, with prior reports around 40.0% [11]). The truncated EML4 domain in V3 may enhance ALK dimerization and MAPK/STAT3 signaling, synergizing with *EGFR*‐driven PI3K/AKT activation to promote tumor survival.

### 4.3 Prognostic Implications

Our analysis identified smoking status as a robust independent predictor of improved OS in both the internal and external cohorts. This dual‐cohort validation underscores smoking history as a clinically actionable stratification factor for patients with *EGFR*‐*ALK*/*ROS1* co‐mutations. The survival advantage in never‐smokers likely reflects two synergistic mechanisms: (1) DNA methylation changes: Tobacco smoke alters methylation patterns, such as causing hypomethylation of detoxification genes like AHRR, which may impair carcinogen metabolism and affect drug‐response pathways [12]; (2) Modulation of cellular pathways: Tobacco smoke disrupts key signaling pathways, including the AMPK/mTOR axis, potentially leading to resistance to TKIs in NSCLC cells [13]. Notably, the female sex showed a prognostic advantage in both cohorts, though statistical significance varied. In the pooled literature cases, females exhibited significantly superior OS compared to males (HR = 3.70, 95% CI: 0.98–13.97, *p* = 0.04), while the internal cohort demonstrated a congruent but non‐significant trend (HR = 4.60, 95% CI: 0.50–42.20, *p* = 0.14) in all co‐mutation cases or in the late‐stage cases (HR = 5.89, 95% CI: 0.64–53.75, *p* = 0.08). This divergence likely reflects sample size limitations (internal vs. external: 17 vs 19). While younger age (≤ 63 years) correlated with improved OS in our internal cohort (*p* = 0.03 for the late‐stage cases), this association was not replicated externally (HR = 0.98, *p* = 0.56). The discordance may reflect differential treatment tolerance thresholds across age groups or unmeasured confounders (e.g., heterogeneous comorbidity management between institutions). Nevertheless, the consistent identification of smoking status as a prognostic determinant across both cohorts highlights its primacy in risk stratification for this molecular subset.

### 4.4 Therapeutic Implications

Therapeutically, our findings suggest that dual‐targeted TKI therapy may be associated with improved outcomes compared with monotherapy or sequential treatment approaches, although this observation requires cautious interpretation due to the limited sample size and retrospective nature of the study. For instance, one *EGFR*‐*ALK* patient treated with a combination of dacomitinib and alectinib survived for over 51 months, and an *EGFR*‐*ROS1* patient receiving gefitinib plus crizotinib achieved stable disease at 3 m before being lost to follow‐up. Importantly, dual‐targeted TKI therapy was generally well tolerated in our cohort, with no clinically significant treatment‐related toxicities observed apart from a single case of cutaneous toxicity that resolved after dose adjustment. Although our sample size is limited, survival curves from both internal and pooled data indicate a trend favoring dual‐targeted therapy. In contrast, patients treated with EGFR‐TKI monotherapy had a median OS approximately 10 m shorter than the 34.2 months previously reported for *EGFR*‐only patients [14], possibly due to compensatory activation of alternative pathways. These findings align with preclinical models showing that simultaneous inhibition of EGFR and ALK delays resistance, although treatment‐related toxicities and the absence of standardized dosing underscore the need for prospective trials [2].

### 4.5 Limitations and Future Directions

The small sample size and retrospective design limit the generalizability of our findings. Molecular profiling was not uniform, with patients undergoing either NGS or multiplex PCR, and NGS testing further varied between 14‐gene and 196‐gene panels. These inconsistencies may have affected mutation detection sensitivity and introduced potential bias in observed co‐mutation frequencies. In addition, due to institutional policy and data access constraints, the NC control group only represents a subset of patients that were derived from the consecutively screened cohort, which may introduce selection bias. We also acknowledge the lack of protein‐level confirmation for some fusion cases due to limited tissue availability, which remains a limitation of our study. Prospective trials with dynamic molecular monitoring comparing sequential versus concurrent targeted therapies are needed to validate efficacy and optimize dosing. Dual‐targeted approaches may counteract resistance mechanisms observed with monotherapy, but limitations in sample size require cautious interpretation. Further studies should address toxicity and refine treatment strategies.

## 5 Conclusion

*De novo EGFR*‐*ALK* and *EGFR*‐*ROS1* co‐mutations define a distinct molecular subset of NSCLC with unique clinical and molecular features. Our findings suggest that patients harboring these alterations may benefit from individualized dual‐targeted therapies, underscoring the importance of comprehensive molecular profiling at diagnosis. Prospective studies with standardized assessments are essential to validate these treatment strategies and improve prognostic evaluations.

## Author Contributions

**Lili Shen:** conceptualization (equal), data curation (equal), formal analysis (equal), investigation (equal), methodology (equal), project administration (equal), resources (equal), software (equal), supervision (lead), validation (equal), visualization (equal), writing – original draft (lead). **Hongyu Deng:** conceptualization (equal), data curation (equal), formal analysis (equal), investigation (equal), methodology (equal), project administration (equal), resources (equal), software (equal), supervision (equal), validation (lead), visualization (equal), writing – original draft (lead). **Wei Liao:** conceptualization (equal), data curation (equal), investigation (equal), methodology (equal), project administration (equal), software (equal), supervision (equal), validation (equal), visualization (equal). **Qiang Gu:** conceptualization (equal), data curation (equal), formal analysis (equal), methodology (equal), supervision (equal), validation (equal), visualization (equal). **Lingling Ma:** data curation (equal), formal analysis (equal), investigation (equal), methodology (equal), validation (equal), visualization (equal). **Qingming Jiang:** conceptualization (equal), data curation (equal), formal analysis (equal), investigation (equal), methodology (equal), project administration (equal), resources (lead), software (equal), supervision (equal), validation (equal), visualization (equal), writing – review and editing (equal). **Kaihua Liu:** conceptualization (equal), data curation (equal), formal analysis (equal), investigation (equal), methodology (equal), project administration (equal), resources (equal), software (equal), supervision (equal), validation (equal), visualization (equal), writing – review and editing (lead).

## Ethics Statement

All participants provided written informed consent for data usage. The study protocol, which includes both co‐mutation analysis and NC control group comparisons, has been approved by the ethics board of Chongqing Cancer Hospital (CZLS2023374‐A).

## Conflicts of Interest

The authors declare no conflicts of interest.

## Supporting information


**Supplementary Figure S1.** Distribution of molecular testing methods in study cohorts.
**Supplementary Figure S2.** Kaplan–Meier survival analysis in the co‐mutation cohort.

**Data S1.** Supplementary Information.

**Supplementary Table S1.** Gene lists for the 14‐gene and 196‐gene NGS panels and AmoyDx 3‐gene assay.

**Supplementary Table S2.** Baseline characteristics of NSCLC patients with EGFR‐ALK and EGFR‐ROS1 co‐mutations.

**Supplementary Table S3.** Integrated clinical and molecular findings from published studies on EGFR‐ALK co‐mutations.

**Supplementary Table S4.** Integrated clinical and molecular findings from published studies on EGFR–ROS1 co‐mutations.

**Supplementary Table S5.** Clinical features of NSCLC with EGFR‐ALK/ROS1 co‐mutations in pooled literature cases.

**Supplementary Table S6.** Cox regression analysis in NSCLC with co‐mutations.

## Data Availability

The original contributions presented in the study are included in the article/Supporting Information. Further inquiries can be directed to the corresponding author.
