# Supplementary material for: De Novo EGFR ‐ ALK and EGFR ‐ ROS1 Co‐Mutations in NSCLC: Clinical Characteristics, Molecular Profiling, and Treatment Outcomes From a Retrospective Analysis
Source: Cancer Med. 2025 Jul 29;14(15):e71084. doi: 10.1002/cam4.71084 (PMC12304520; doi:10.1002/cam4.71084)
Supplement: Supplementary file 1 — Supplementary Figure S1. Distribution of molecular testing methods in study cohorts. Supplementary Figure S2. Kaplan–Meier survival analysis in the co‐mutation cohort. [file CAM4-14-e71084-s002.docx]

**Supplementary Figures**

**[Supplementary Figure S1](#_Toc201822125)** [1](#_Toc201822125)

**[Supplementary Figure S2](#_Toc201822126)** [3](#_Toc201822126)

**Supplementary Figure S1**


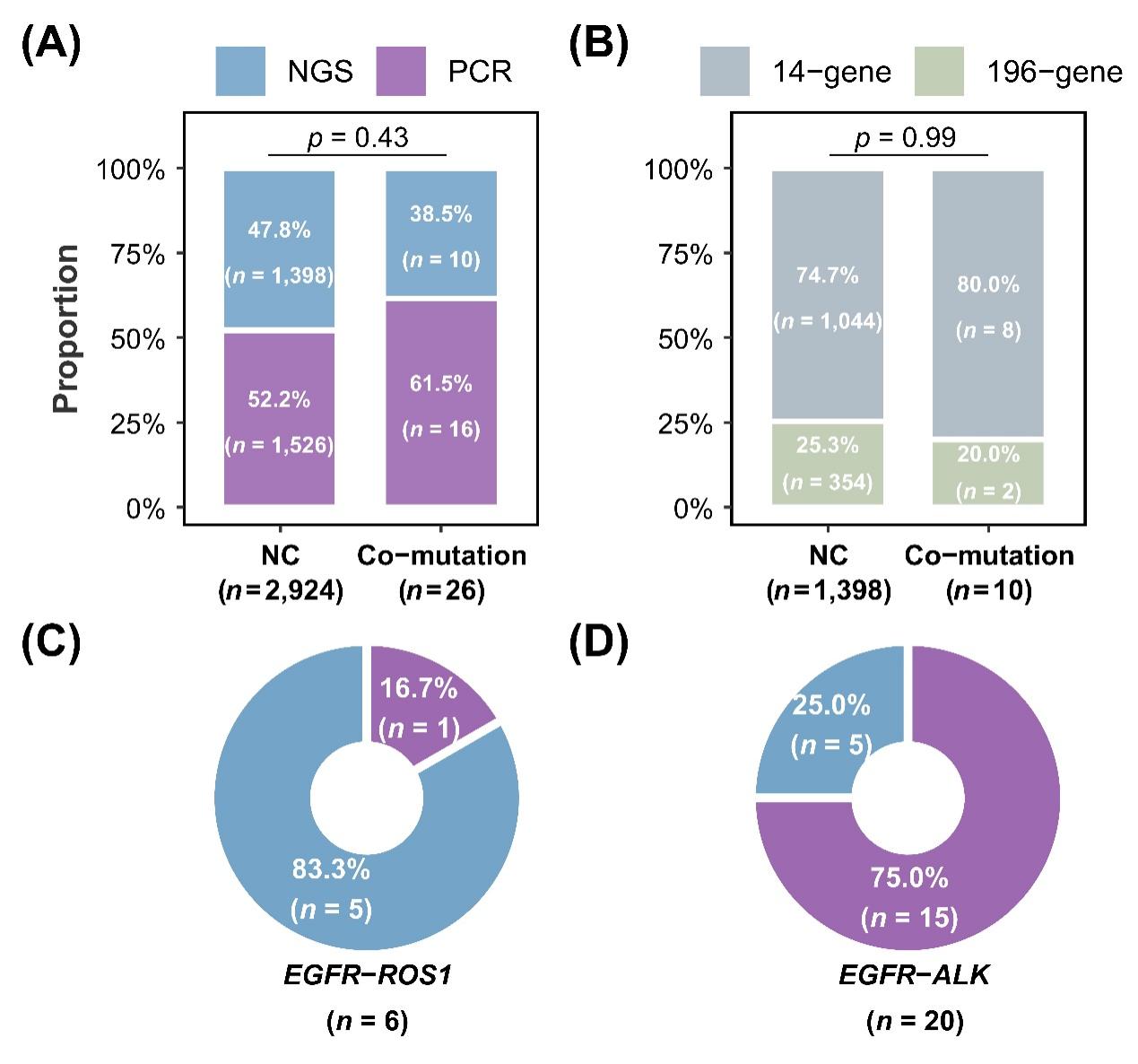


**Supplementary Figure S1. Distribution of molecular testing methods in study cohorts.**

(A) Stacked bar plot displaying the proportion of patients tested by NGS or multiplex PCR in the NC cohort and the co-mutation cohort. Percentages and patient counts are indicated within each segment of the bars. Different colors represent NGS and multiplex PCR, respectively.

(B) Stacked bar plot displaying the proportion of patients tested by different NGS panels in the NC cohort and the co-mutation cohort. Percentages and patient counts are indicated within each segment of the bars. Different colors represent the 14-gene panel and the 196-gene panel, respectively.

NGS, next-generation sequencing; PCR, polymerase chain reaction; NC, Non-Co-mutated; EGFR, epidermal growth factor receptor; ROS1, ROS proto-oncogene 1; ALK, anaplastic lymphoma kinase.

**Supplementary Figure S2**


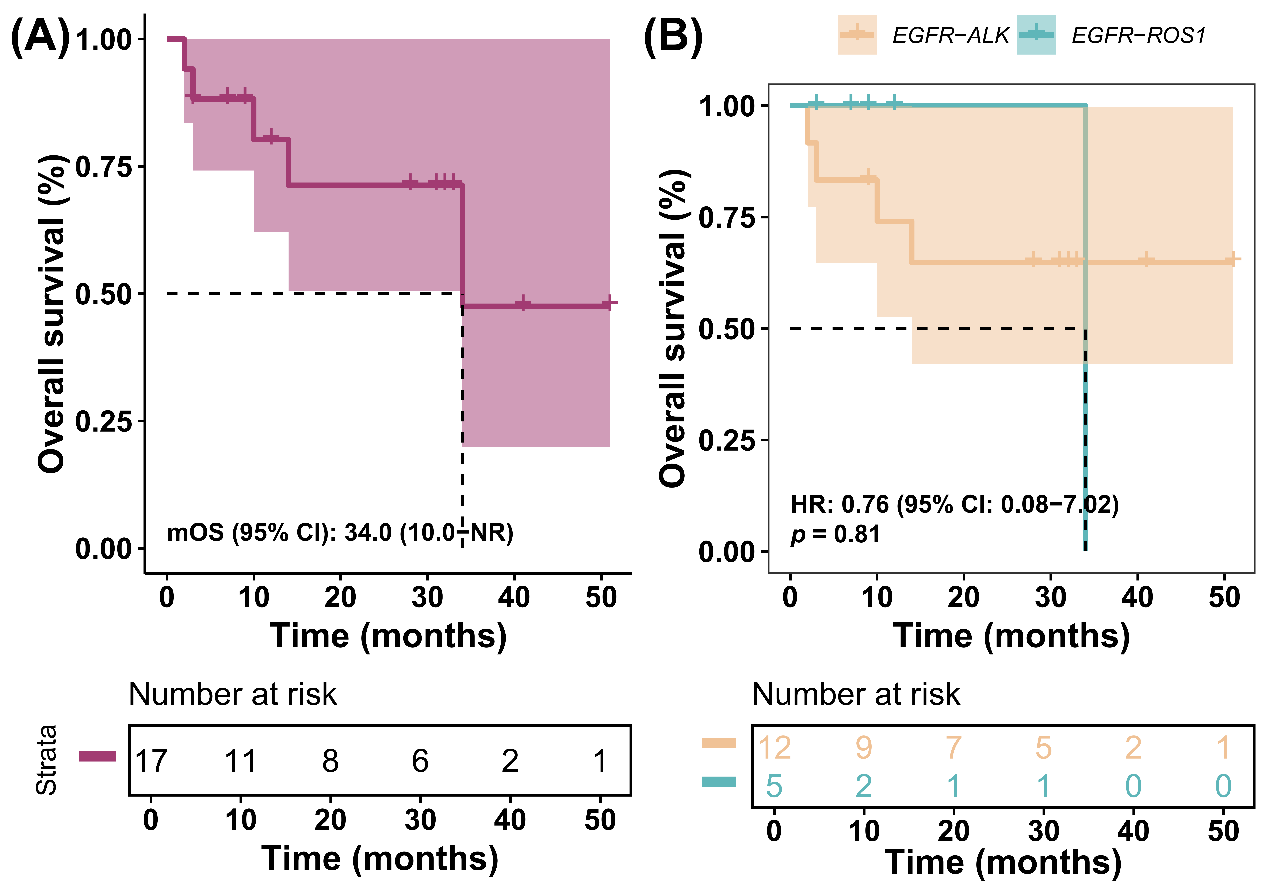


**Supplementary Figure S2. Kaplan–Meier survival analysis in the co-mutation cohort.**

**(A)** Kaplan-Meier curve showing the OS of 17 patients in the co-mutation cohort with available OS data. Median OS and 95% confidence interval are indicated on the plot.

**(B)** Kaplan-Meier curves comparing the OS between the *EGFR*-*ALK* and *EGFR*-*ROS1* subgroups within the co-mutation cohort. Hazard ratio, 95% CI, and *p*-value are displayed on the plot.

OS, overall survival; mOS, median overall survival; CI, confidence interval; HR, hazard ratio; EGFR, epidermal growth factor receptor; ROS1, ROS proto-oncogene 1; ALK, anaplastic lymphoma kinase.
