# Supplementary material for: De Novo EGFR ‐ ALK and EGFR ‐ ROS1 Co‐Mutations in NSCLC: Clinical Characteristics, Molecular Profiling, and Treatment Outcomes From a Retrospective Analysis
Source: Cancer Med. 2025 Jul 29;14(15):e71084. doi: 10.1002/cam4.71084 (PMC12304520; doi:10.1002/cam4.71084)
Supplement: Supplementary file 2 — Data S1. Supplementary Information. [file CAM4-14-e71084-s004.docx]

**DNA extraction and targeted NGS**

For patients with surgical specimens, tumor tissues were first inspected by pathologists to ensure that they contained at least 20% tumor content and sufficient sample for testing. Tissue DNA was extracted with the QIAamp DNA FFPE Tissue kit (Qiagen, Germantown, MD, USA). Quantity and quality of the extracted DNA were evaluated by Qubit 3.0 fluorometer and Nanodrop 2000, respectively (Thermo Fisher Scientific). Library preparations were performed using KAPA Hyper Prep kit (KAPA Biosystems) following manufacturer’s protocol. Hybridization-based target enrichment was carried out using the GeneseeqPrime® pan-cancer gene panel with xGen Lockdown Hybridization and Wash Reagents Kit (Integrated DNA Technologies). Captured libraries by Dynabeads M-270 (Life Technologies) were amplified in KAPA HiFi HotStart ReadyMix (KAPA Biosystems) and quantified by qPCR using KAPA Library Quantification Kit (KAPA Biosystems). The target enriched library was then sequenced on the NextSeq550Dx NGS platform (Illumina) following the manufacturer’s instructions.

**Sequence alignment and data processing**

Trimmomatic was used for FASTQ file quality control. Leading/ trailing low quality (below 20) or N bases were removed. The sequencing data was aligned to the reference Human Genome (hg19) using Burrows-Wheeler Aligner (BWA-mem, v0.7.12). Alignment results underwent de-duplication by Sambamba (1). Base quality recalibration and indel realignment were processed by Genome Analysis Toolkit (GATK 3.4.0). VarScan2 was employed for calling single-nucleotide variations (SNVs) and insertion/deletions (Indels) which were identified with a minimum variant allele frequency threshold set at 0.01 and p value threshold for calling variants set at 0.05 to generate Variant Call Format files. All SNVs/indels were annotated with ANNOVAR. The sequencing assay has been validated in compliance with college of American pathologists (CAP) and clinical laboratory improvement amendments (CLIA) with a limit of detection of 1% VAF for tissue. Genomic fusions were identified by FACTERA with default parameters. Copy number variations (CNVs) were detected using CNVkit with default parameters. Depth ratios of above 2.0 (tissue) and below 0.6 were considered as CNV gain and CNV loss, respectively.

1. Tarasov A, Vilella AJ, Cuppen E, Nijman IJ, Prins P. Sambamba: fast processing of NGS alignment formats. Bioinformatics. 2015;31(12):2032-4.
